# Supplementary material for: Tuning CAR-T cells by targeting cancer-associated glycan in pancreatic cancer
Source: Nat Commun. 2025 Dec 10;16:11246. doi: 10.1038/s41467-025-66102-2 (PMC12717153; doi:10.1038/s41467-025-66102-2)
Supplement: Supplementary file 1 — Supplementary Information [file 41467_2025_66102_MOESM1_ESM.pdf]

This Supplementary Information file contains:

- Supplementary Figures 1-12

- Supplementary Tables 1-3

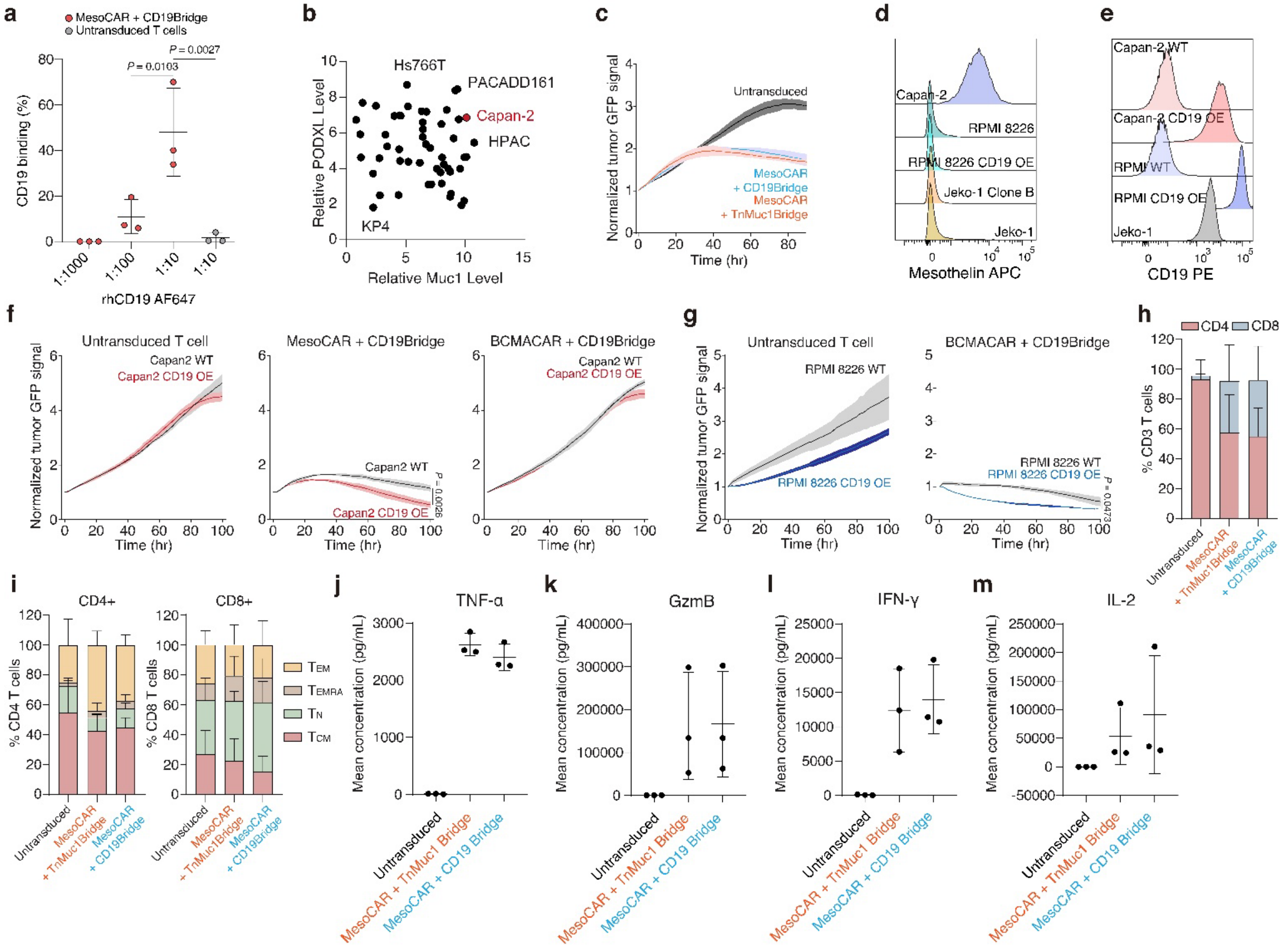

Supplementary Figure 1. Mesothelin-targeted CAR-T cells with CD19 bridge system. **a**, Flow cytometry analysis of Alexa Fluor 647 conjugated-recombinant CD19 levels on CD19 bridge T cells after 30 minutes incubation at 37°C. Results are mean ± s.d. of n = 3 distinct human blood donors **b,** Representative mucins (e.g. podocalyxin (PODXL) and Muc1) transcript levels as measured by the DepMap project (n=52 cell lines). **c**, Real-time cytotoxicity assay against Capan-2 cells with Mesothelin CAR-T cells with Tn-Muc1 bridge or CD19 bridge at a 0.5:1 E:T ratio (relative to day 0 tumor seeding). Results are mean ± s.d. of n = 3 independent measurements. **d,e**, Flow cytometry analysis of mesothelin (**c**) and CD19 (**d**) surface levels on indicated cancer cell lines. **f,g**, Representative real-time cytotoxicity assay against Capan-2 wild-type, Capan-2 CD19 OE (**e**) or RPMI 8226 wild-type, RPMI 8226 CD19 OE (**f**) at a 1:1 E:T ratio (relative to day 0 tumor seeding) from n = 3 human blood donors. Results are mean ± s.d. of n = 3 independent measurements. **h,i**, CD4 and CD8 population (**h**) and phenotype (**i**) of CAR-T cells with Tn-MUC1 bridge or CD19-bridge and untransduced T cells. Cells were grouped by flow cytometry according to T-cell phenotypes as follows: naïve (T_N): $CCR7^+CD45RO^-$, T_{CM}: $CCR7^+CD45RO^+$, T_{EM}: $CCR7^-CD45RO^+$ and effector (T_E): $CCR7^-CD45RO^-$. Results are mean ± s.d. of n = 3 distinct human blood donors. **j-m**, Secreted cytokine levels after coculture with Capan-2 *C1GALT1* KO and indicated CAR-T cells at a 1:1 E:T ratio. Results are mean ± s.d. of n = 3 distinct human blood donors. In **d,e**, representative flow cytometry data from three independent experiments. In **a**, statistical analysis was performed by one-way ANOVA with Tukey's post hoc tests. In **f** and **g**, statistical analysis was performed by two-way ANOVA with correction for multiple comparisons.

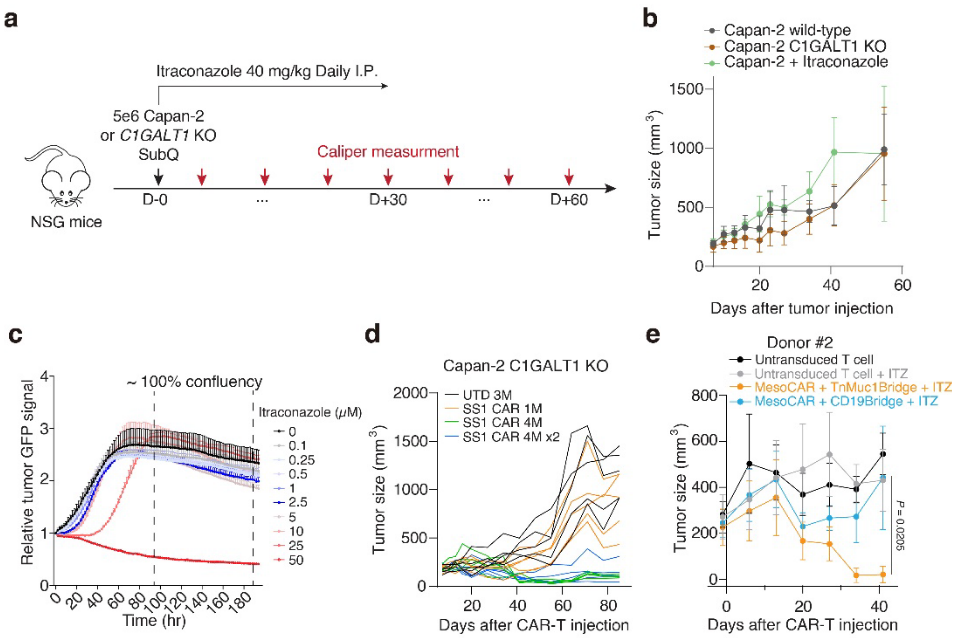

Supplementary Figure 2. Effects of itraconazole treatment on Capan-2 cell line. **a,b,** NOD.Cg-Prkdc^{scid} Il2rg^{tm1Wjl}/SzJ (NSG) mice were subcutaneously (SubQ) injected with 5 x 10^6 Capan-2 or Capan-2 *C1GALT1* KO cells on day 0 (**a**). The mice were further treated intraperitoneally (I.P.) with itraconazole (40 mg/kg) once daily for 30 days. Tumor growth curve for each mouse in the indicated groups (n = 5 mice per group) (**b**). **c,** Representative real-time cancer cell growth assay of Capan-2 wild-type cells cultured in media containing the indicated itraconazole concentration. Results are mean ± s.d. of n = 3 independent measurements. **d**, NSG mice were subcutaneously injected with 5 x 10^6 Capan-2, Capan-2 *C1GALT1* KO cells on day -14. On Day 0, 5 mice per group received the indicated dose of mesothelin-targeted CAR T cells intravenously. **e,** NSG mice were subcutaneously injected with 5 x 10^6 Capan-2 cells on day -14. On Day 0, 3-5 mice per group received 2.5 x 10^6 CAR T cells with itraconazole treatment. CAR-T cells are the indicated mesothelin-targeted CARs with glyco-bridges or untransduced T cells from distinct human blood donors, as shown in **Fig. 2f**. Results are mean ± s.e.m. of n=5 mice. In **e**, statistical analysis was performed by two-way ANOVA with correction for multiple comparisons.

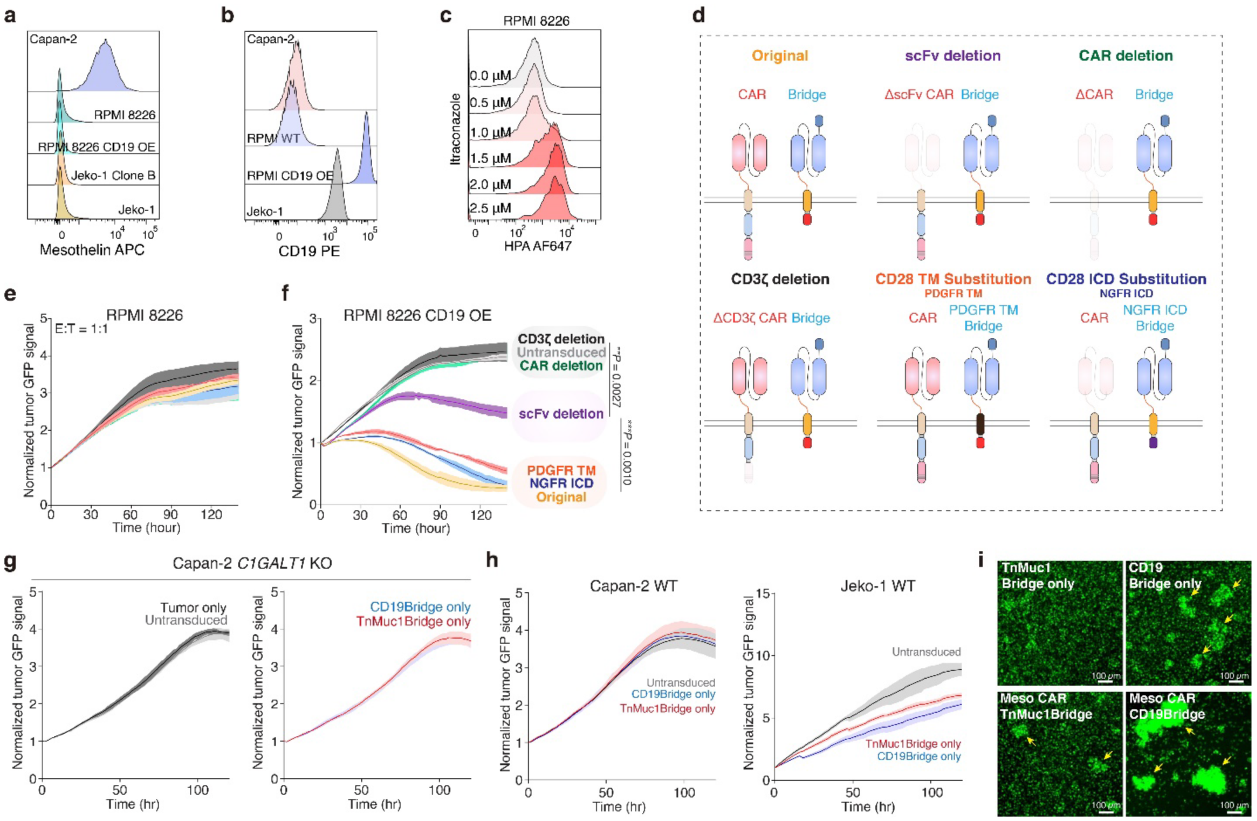

Supplementary Figure 3. Glyco-bridge induces CAR-mediated killing. a,b, Flow cytometry analysis of mesothelin (**a**) and CD19 (**b**) surface levels on indicated cancer cell lines. Data shown are from the same experiment as in **Supplementary Fig. 1c,d**. **c**, Flow cytometry analysis of soluble, fluorophore-labeled HPA lectin binding on RPMI 8226 cell line after treatment with the indicated itraconazole concentration for 48 hours. **d**, Schematic showing the mutation library on CAR and bridge molecules. **e,f**, Real-time cytotoxicity assay against RPMI 8226 (e) and RPMI 8226 overexpressing CD19 (f) cells with the mutation library at a 1:1 E:T ratio (relative to day 0 tumor seeding). Results are mean ± s.d. of n = 3 independent measurements. **g**, Real-time cytotoxicity assay against Capan-2 *C1GALT1* KO cells with only Tn-Muc1 bridge or CD19 bridge at a 1:1 E:T ratio (relative to day 0 tumor seeding). Results are mean ± s.d. of n = 3 independent measurements. **h**, Real-time cytotoxicity assay against Capan-2 (left) and Jeko-1 (right) with ΔSS1 CAR-T cells with the indicated bridges at a 1:1 E:T ratio (relative to day 0 tumor seeding). Results are mean ± s.d. of n = 3 independent measurements. **i**, Representative fluorescent images of mesothelin-overexpressing Jeko-1 GFP cells after coculturing with indicated CAR-T cells from **Fig. 3i** and **h** (n = 3 independent measurements). Scale bar, 100 µm. In **a-c**, representative flow cytometry data from three independent experiments. In **c**, statistical analysis was performed by two-way ANOVA with correction for multiple comparisons.

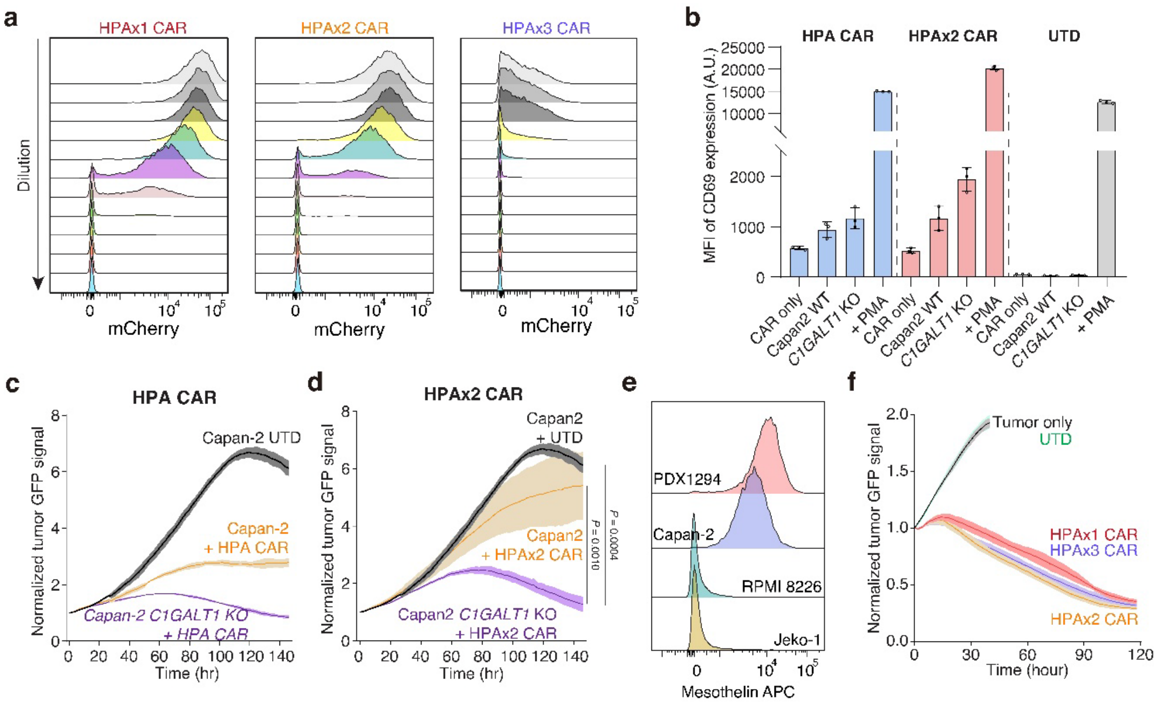

Supplementary Figure 4. HPA-based CAR-T cells against Capan2 *C1GALT1* KO cells and PDX1294 cells. a, Flow cytometry analysis of lentiviral transduction efficiency with indicated HPA-based CAR constructs. **b**, Flow cytometry analysis of CD69 expression on CAR T cells followed by incubation with the indicated target cells for 24 hours. Results are mean ± s.d. of n = 3 technical replicates. PMA-ionomycin was used as a positive control. **c-d**, Real-time cytotoxicity assay against Capan-2 and Capan2 *C1GALT1* KO with HPAx1-based CAR-T cells (**c**) or HPAx2-based CAR-T cells (**d**) at a 1:1 E:T ratio (relative to day 0 tumor seeding). Results are mean ± s.d. of n = 3 independent measurements. **e**, Flow cytometry analysis of mesothelin surface levels on indicated cancer cell lines. Data shown are from the same experiment as in **Supplementary Fig. 1c, 3a. f,** Real-time cytotoxicity assay against PDX1294 cells with indicated HPA-based CAR-T cells at a 0.5:1 E:T ratio (relative to day 0 tumor seeding). Results are mean ± s.d. of n = 3 independent measurements. In **e**, representative flow cytometry data from three independent experiments. In **c-d,** statistical analysis was performed by two-way ANOVA with correction for multiple comparisons.

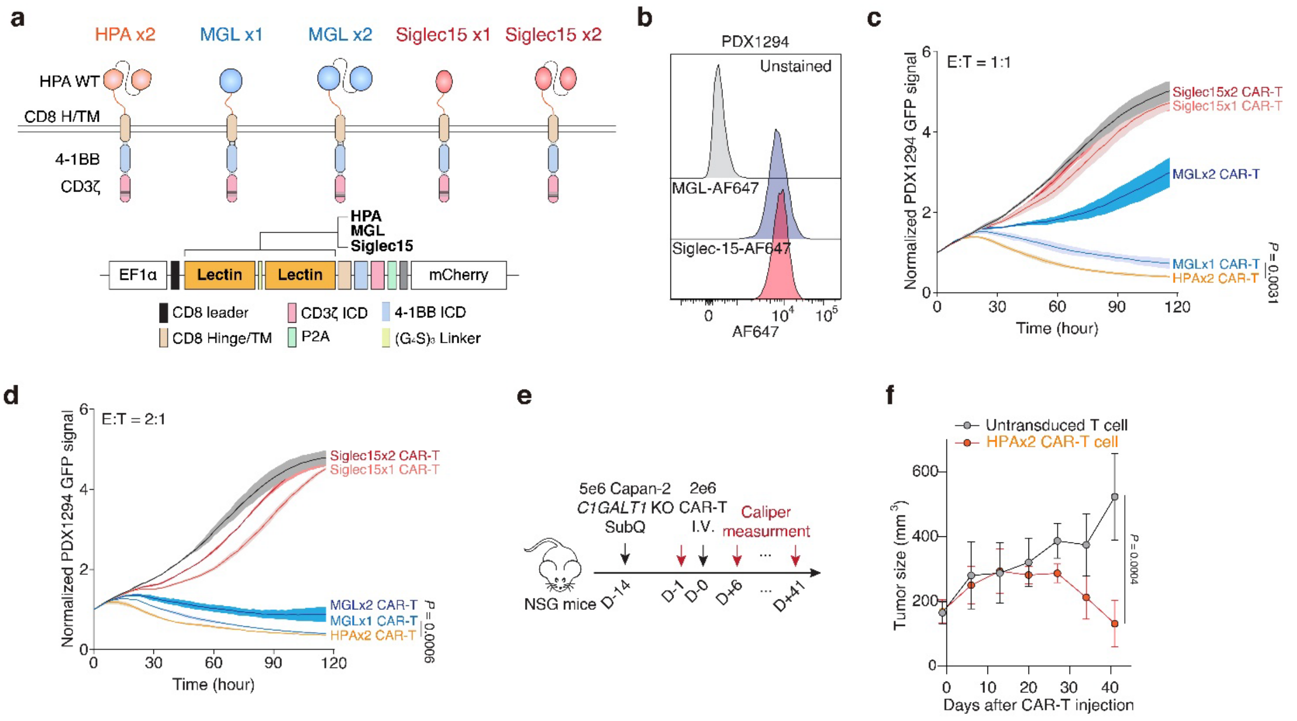

Supplementary Figure 5. Tn antigen-targeted CAR-T cells against PDX1294 cells and Capan-2 *C1GALT1* KO. **a**, Schematic showing Tn antigen-targeted lectin CAR T cells; human macrophage galactose N-acetyl-galactosamine (GalNAc) specific lectin (MGL) and sialic acid-binding immunoglobulin-like lectin (Siglec-15) were used to compare with a dual HPA-based CAR T cells. **b**, Flow cytometry analysis of fluorescent-labelled recombinant MGL and Siglec15 binding to PDX1294 cells. **c-d**, Real-time cytotoxicity assay against PDX1294 with indicated CAR-T cells at a 1:1 (**c**) or 2:1 (**d**) E:T ratio (relative to day 0 tumor seeding). Results are mean ± s.d. of n = 3 independent measurements. **e**, NSG mice were subcutaneously injected with 5 x 10^6 Capan-2 *C1GALT1* KO cells on day -14. On Day 0, 5 mice per group received 2 x 10^6 UTD or HPAx2 CAR T cells intravenously. **f**, Tumor growth curve for each condition (n = 5 mice). Results are mean ± s.e.m. of n=5 mice. In **b**, representative flow cytometry data from three independent experiments In **c-d,f,** statistical analysis was performed by two-way ANOVA with correction for multiple comparisons.

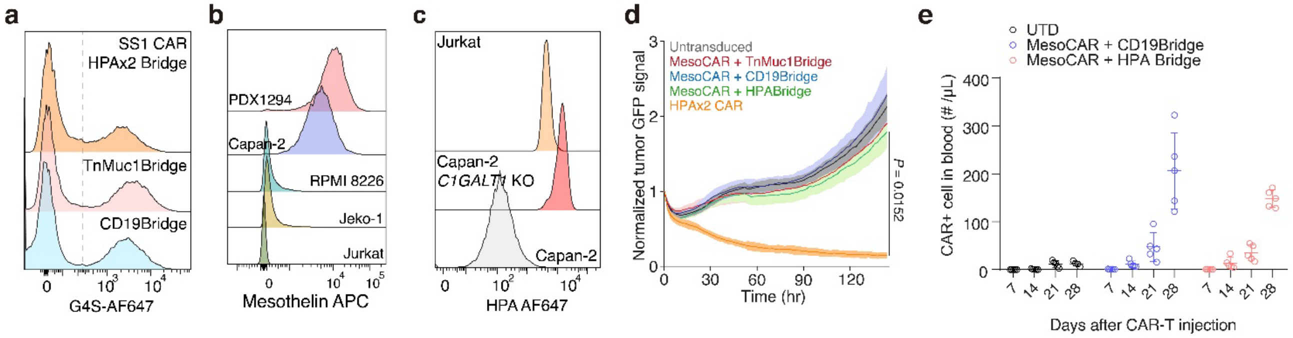

Supplementary Figure 6. CAR-T cells with HPA-based bridge exhibit reduced bridge-mediated killing and enhanced cell avidity. a, Flow cytometry analysis of $(G_4S)_3$ linkers expression levels on T cells. **b,c**, Flow cytometry analysis of mesothelin (**b**) and Tn antigen (**c**) expression of Jurkat and indicated cell lines. Data shown are from the same experiment as in **Supplementary Fig. 1c, 3a, 4e**. **d**, Real-time cytotoxicity assay against Jurkat cells with indicated CAR-T cells at a 1:1 E:T ratio (relative to day 0 tumor seeding). Results are mean ± s.d. of n = 3 independent measurements. **e**, Absolute count of CAR-T cells in the peripheral blood at 7-, 14-, 21-, and 28-days post CAR-T cell injection. In **a-c**, representative flow cytometry data from three independent experiments In **d**, statistical analysis was performed by two-way ANOVA with correction for multiple comparisons.

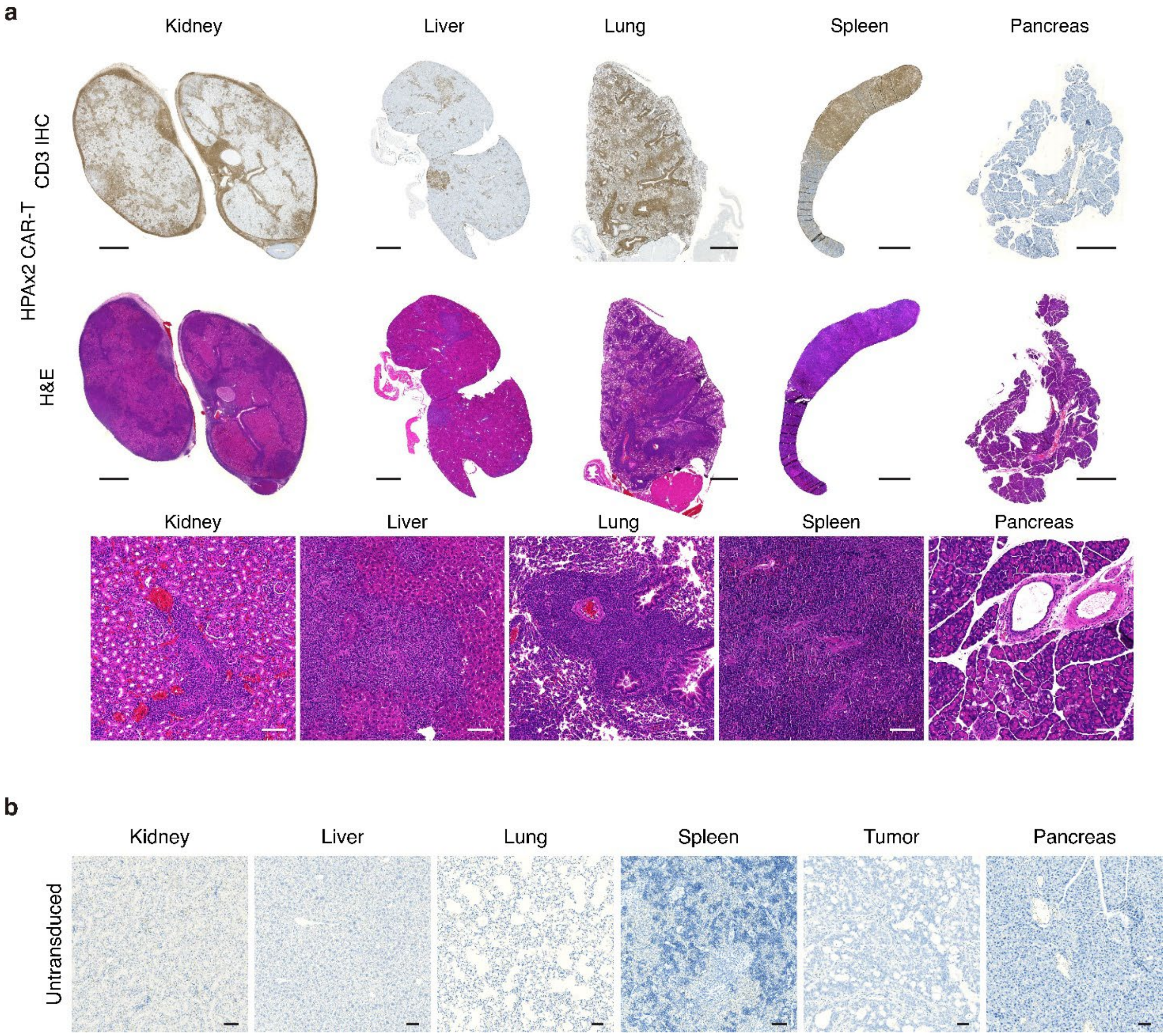

Supplementary Figure 7. Toxicity of HPA-based CAR-T cell and HPA-based bridge approach. a, Representative IHC images for human CD3 staining and H&E staining in formalin-fixed, paraffin-embedded specimens from mice injected with Capan-2 *C1GALT1* KO cells and HPAx2 CAR-T cells from **Fig. 4n** (n=5 mice). Scale bar, 1 mm (Top) and 100 µm (Bottom). **b**, Representative IHC images for human CD3 staining comparing UTD across different organs from **Fig. 5g-m** (n=5 mice). Scale bar, 100 µm

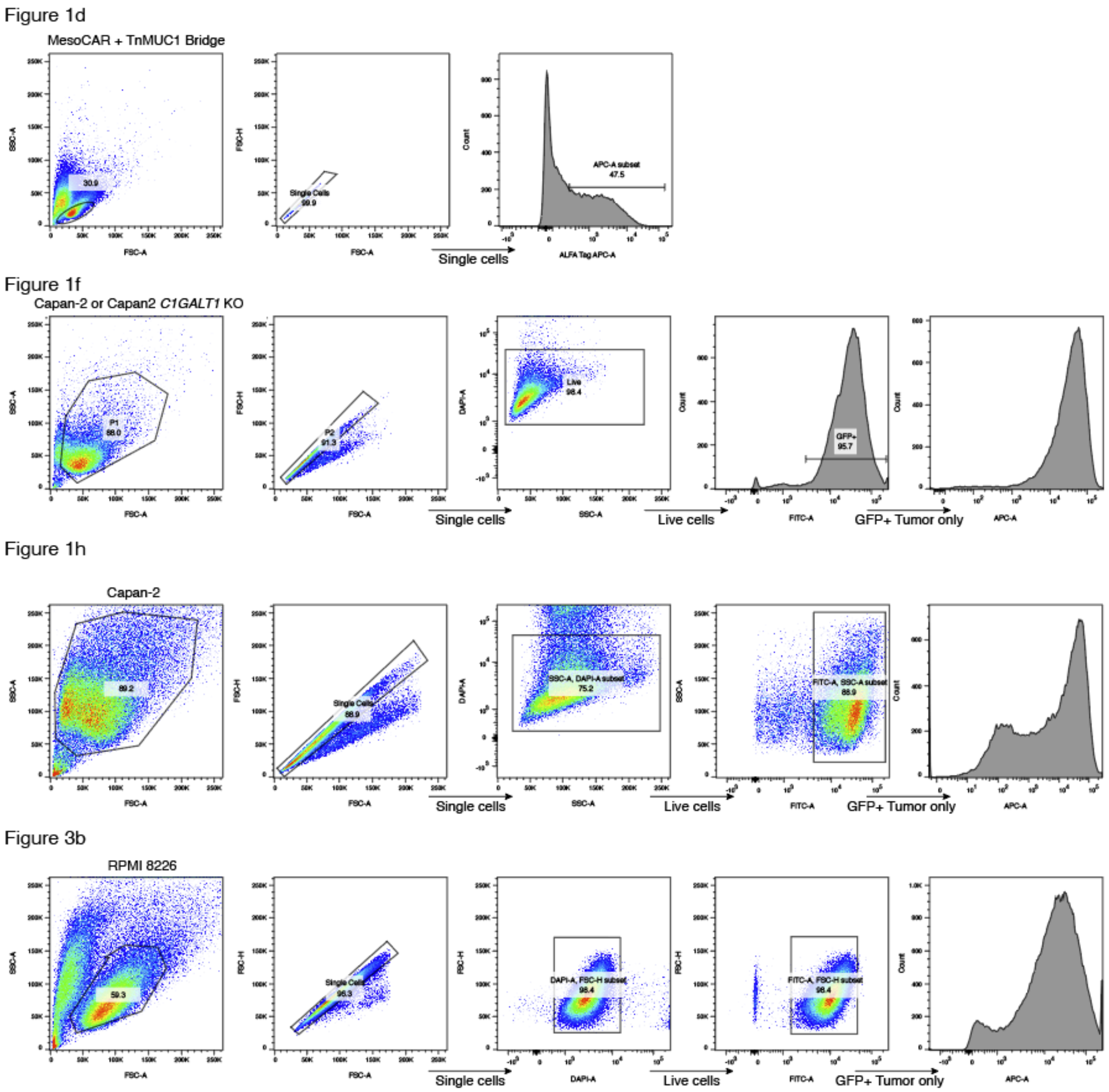

Supplementary Figure 8. FACS sequential gating/sorting strategies for Fig. 1-3b

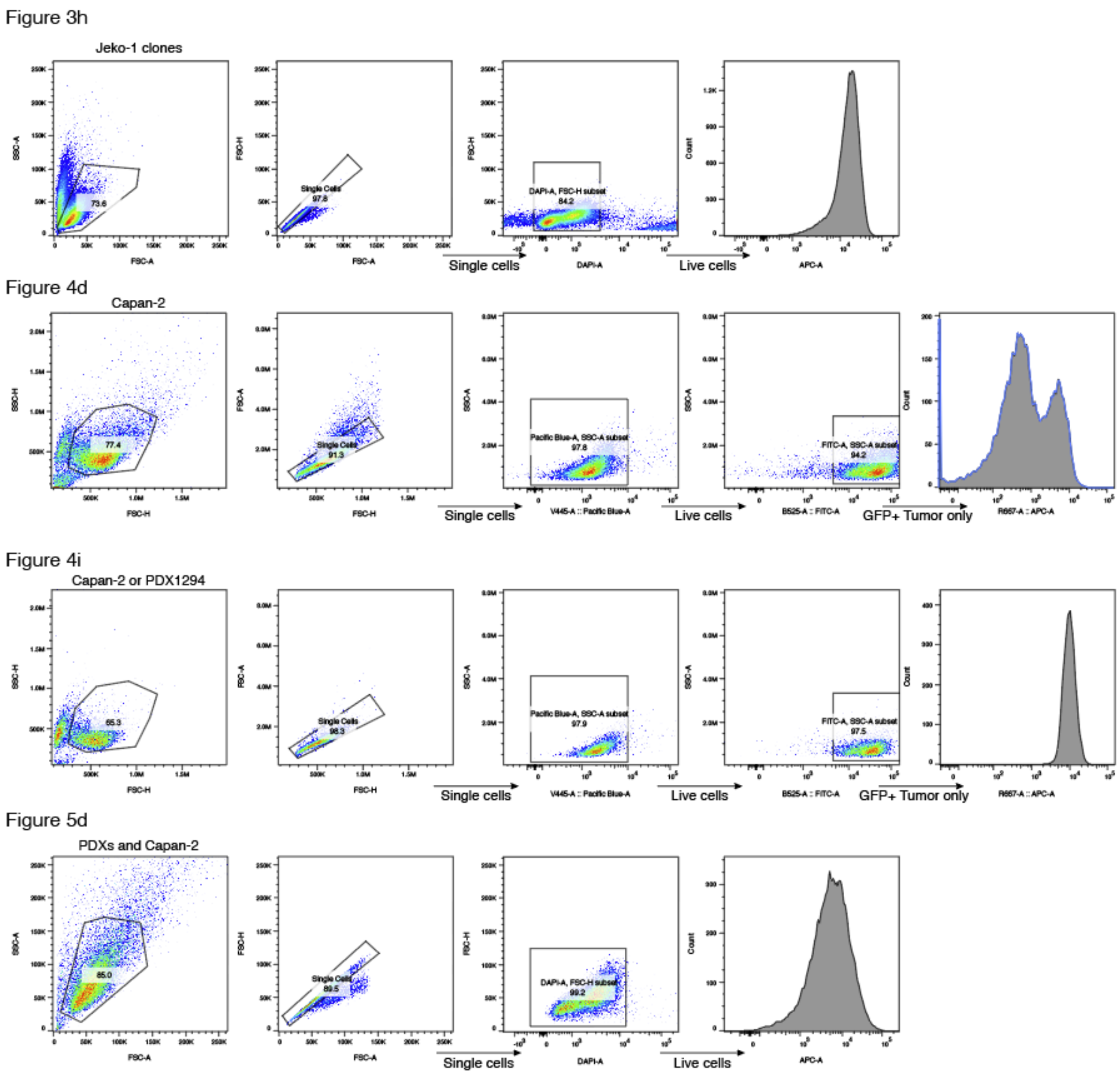

Supplementary Figure 9. FACS sequential gating/sorting strategies for Fig. 3h-5d

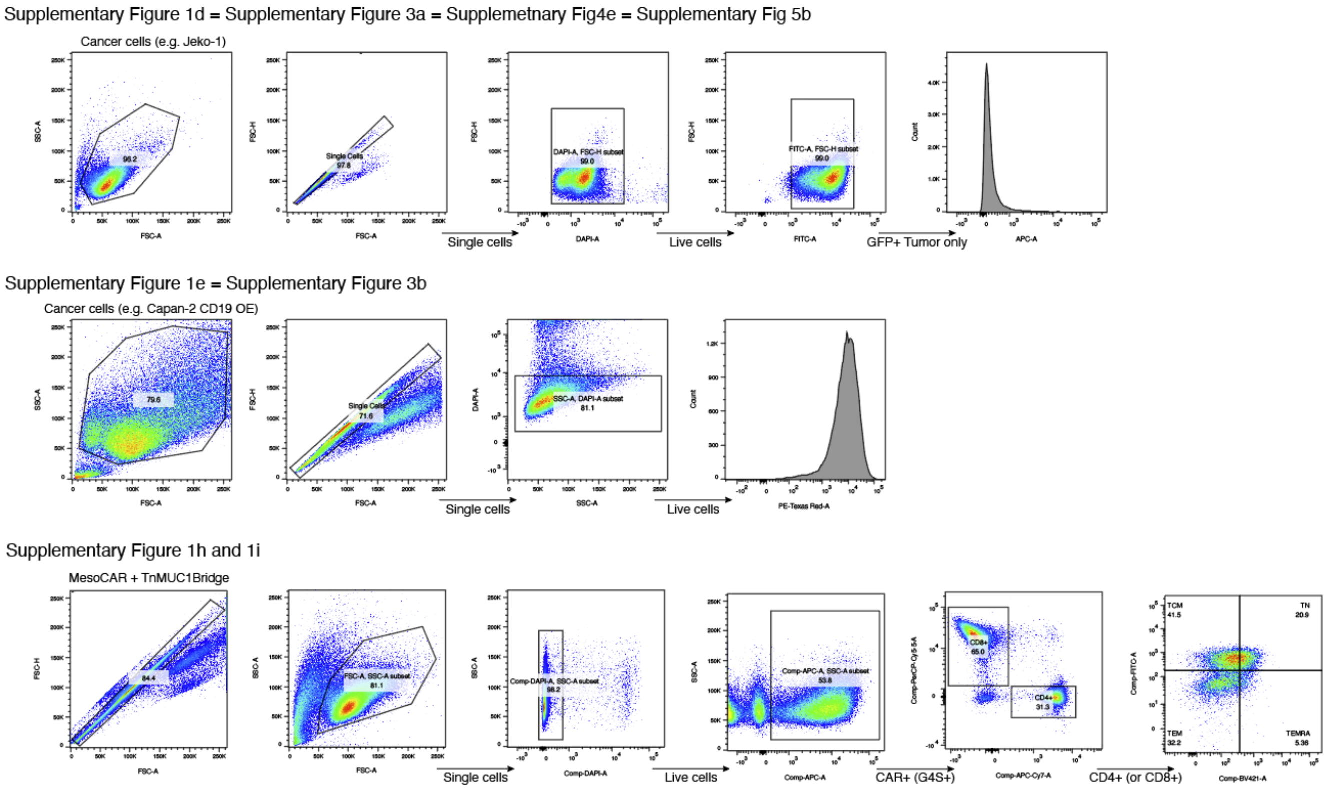

Supplementary Figure 10. FACS sequential gating/sorting strategies for Supplementary Fig. 1

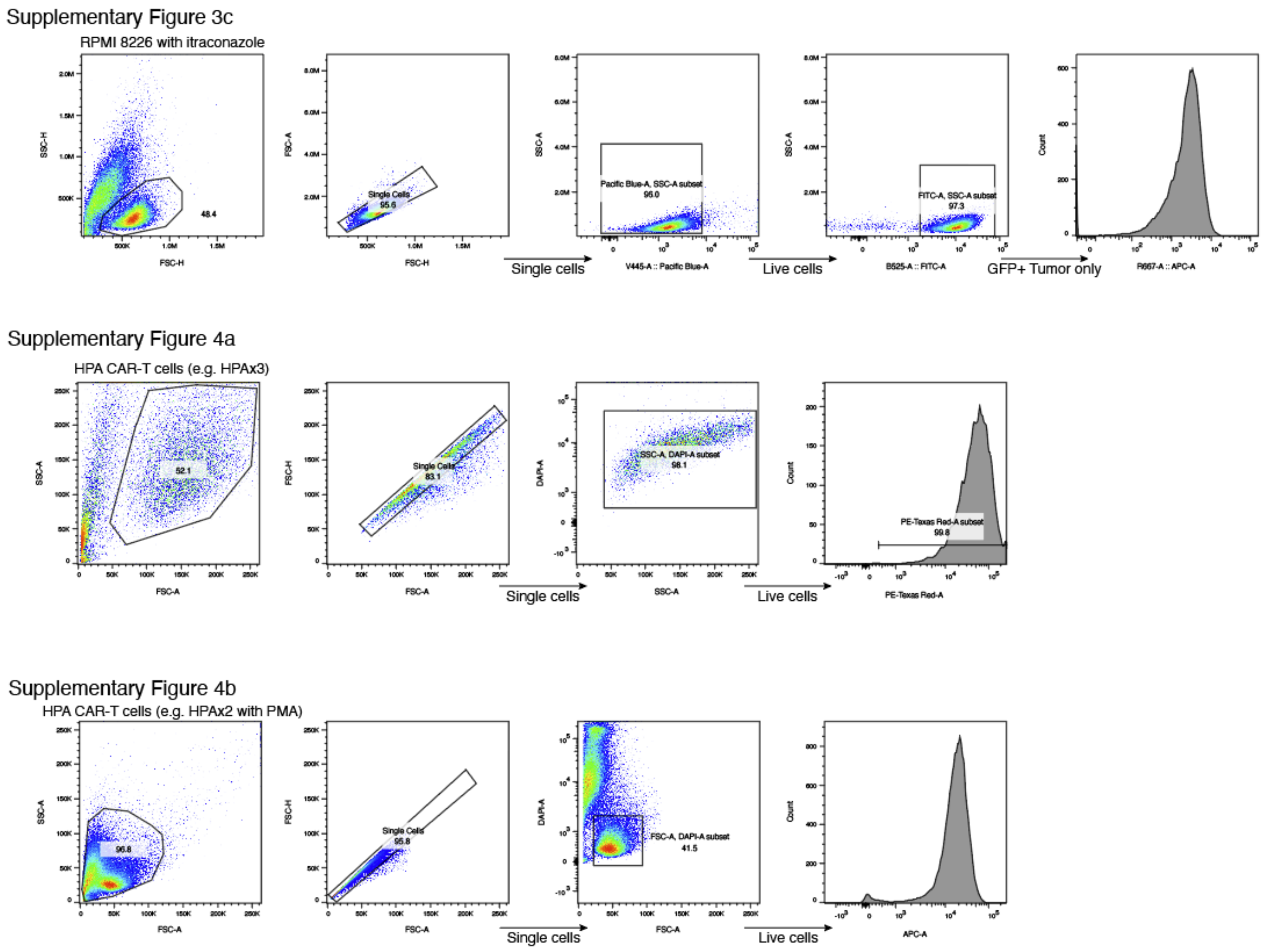

Supplementary Figure 11. FACS sequential gating/sorting strategies for Supplementary Fig. 3,4

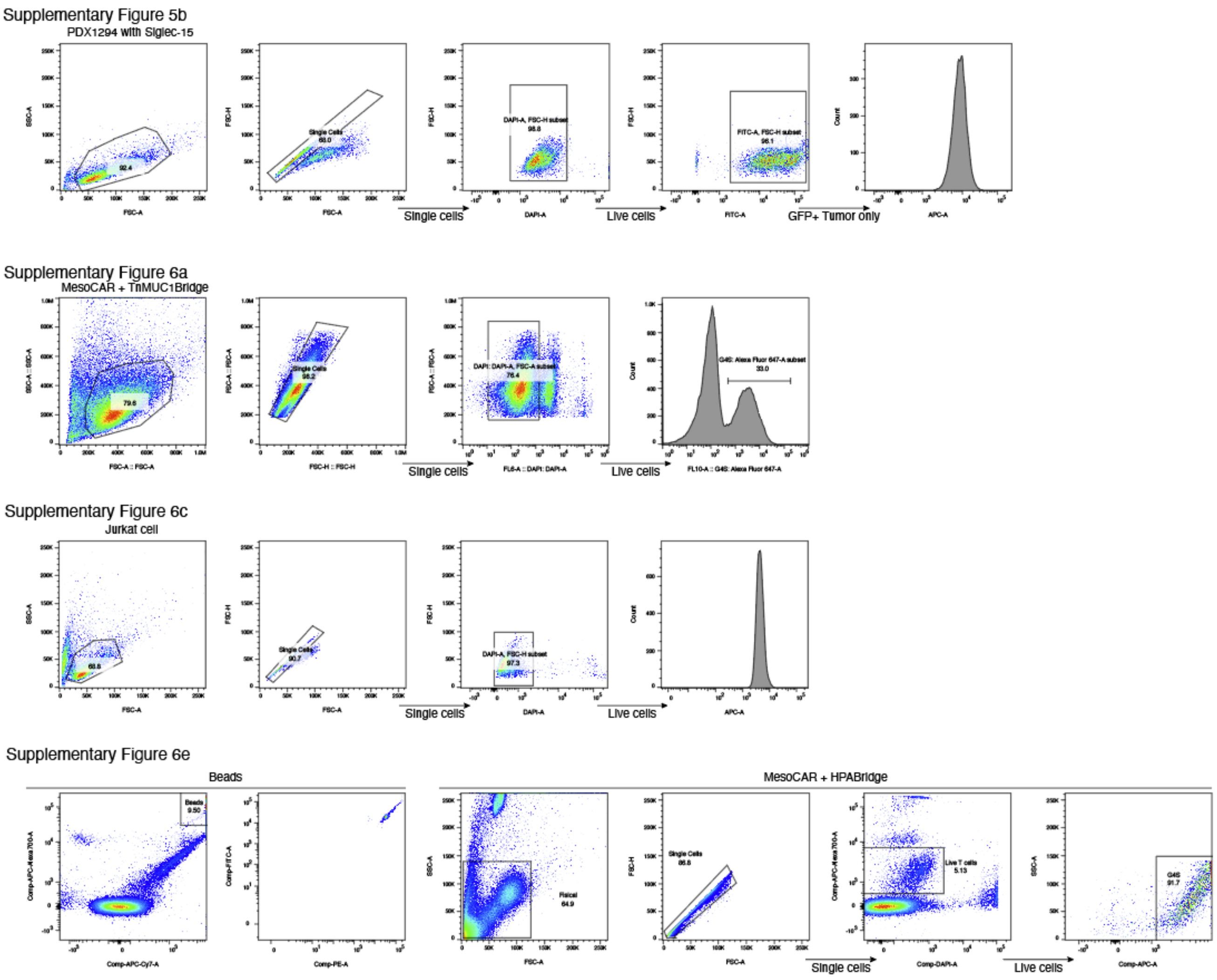

Supplementary Figure 12. FACS sequential gating/sorting strategies for Supplementary Fig. 5,6

Supplementary Table 1. PCR primer sequence and CRISPR RNA sequence for cloning

Constructs	Primers and gBlock
ΔCAR for Mesothelin CAR with Tn-Muc1 bridge	5’-CAGGTGCAGCTGCAGCAGTCTG-3’<br>5’-GGGCCGAGCGGCGTGGAG-3’
ΔCAR for Mesothelin CAR with CD19 bridge	5’-GAAATTGTGATGACCCAGTCACCCGCC-3’<br>5’-GGGCCGAGCGGCGTGGAG-3’
ΔscFv for Mesothelin CAR with Tn-Muc1 bridge and CD19 bridge	5’-ACCACTACCCCAGCACCGAGG-3’<br>5’-GGGCCGAGCGGCGTGGAG-3’
ΔCD3ζ for Mesothelin CAR with Tn-Muc1 bridge and CD19 bridge	5’- TCCGGAGGTTCTGGTGCC -3’<br>5’- AGTTCGCAGCCGCCTTCC -3’
NGFR ICD for Mesothelin CAR with CD19 bridge	5’- cggggcatcctggcttctTAAGTCGACAATCAACCTC -3’<br>5’- gttccaccgcttgctgcgGACCCAGAAGATAATAAACG -3’
PDGFRβ TM for Mesothelin CAR with Tn-Muc1 bridge	5’-CGCAGCAAGCGGAGCCGG-3’<br>5’-CTTCAGTTCCAGCTTGGTGCC-3’<br>gBlock 5’-<br>GCACCAAGCTGGAACTGAAGGCTGTGGGCCAGGACACGC<br>AGGAGGTCATCGTGGTGCCACACTCCTTGCCCTTTAAGGTG<br>GTGGTGATCTCAGCCATCCTGGCCCTGGTGGTGCTCACCAT<br>CATCTCCCTTATCATCCTCATCATGCTTTGGCAGAAGAAGCC<br>ACGTCGCAGCAAGCGGAGCCGGCT-3’
PDGFRβ TM for Mesothelin CAR with CD19 bridge	5’-CGCAGCAAGCGGAGCCGG-3’<br>5’-GCTGGACACGGTGACCAGAGTAC-3’<br>gBlock 5’-<br>CTCTGGTCACCGTGTCCAGCGCTGTGGGCCAGGACACGCA<br>GGAGGTCATCGTGGTGCCACACTCCTTGCCCTTTAAGGTGG<br>TGGTGATCTCAGCCATCCTGGCCCTGGTGGTGCTCACCATC<br>ATCTCCCTTATCATCCTCATCATGCTTTGGCAGAAGAAGCC<br>ACGTCGCAGCAAGCGGAGCCGGCT-3’

Supplemental Table 2. Complete sequence for Mesothelin CAR with Glyco-Bridge

Mesothelin CAR with Glyco-Bridge includes two CAR constructs:

1) Mesothelin CAR or conventional CAR: SS1 scFv, CD8 H/TM, 4-1BB, CD3ζ.
2) Glyco-Brdige: TnMuc1 scFv, CD19 scFv or a dual HPA lectin, CD28 H/TM, inactivated CD28 ICD

TnMuc1 Bridge<br><br>TnMuc1 (5E5) scFv<br><br>CD28 H/TM<br><br>Inactivated CD28 ICD	QVQLQQSDAELVKPGSSVKISCKASGYTFTDHAIHWVKQKPEQGLEWIGHFSPG<br>NTDIKYNDKFKGKATLTVDRSSSTAYMQLNSLTSEDSAVYFCKTSTFFFDYWGQ<br>GTTLTVSSGGGGSGGGGSGGGGSELVMTQSPSSLTVTAGEKVTMICKSSQSLLN<br>SGDQKNYLTWYQQKPGQPPKLLIFWASTRESGVPDRFTGSGSGTDFTLTISSVQA<br>EDLAVYYCQNDYSYPLTFGAGTKLELKIEVMYPPPYLDNEKSNGTIIHVKGKHL<br>CPSPLFPGPSKPFWVLVVVGGVLACYSLLVTVAFIIFWVRSKRSRLLHSDFMNMT<br>PRRPGPTRKHYQAYAAPRDFAAYRS*
CD19 Bridge<br><br>CD19 scFv<br><br>CD28 H/TM<br><br>Inactivated CD28 ICD	EIVMTQSPATLSLSPGERATLSCRASQDISKYLNWYQQKPGQAPRLLIYHTSRLH<br>SGIPARFSGSGSGTDYTLTISSLQPEDFAVYFCQQGNTLPYTFGQGTKLEIKGGGG<br>SGGGGSGGGGSGGGGSQVQLQESGPGLVKPSETLSLTCTVSGVSLPDYGVSWIR<br>QPPGKGLEWIGVIWGSETTYYQSSLKSRVTISKDNSKNQVSLKLSSVTAADTAV<br>YYCAKHYYYGGSYAMDYWGQGTLVTVSSIEVMYPPPYLDNEKSNGTIIHVKGK<br>HLCPSPLFPGPSKPFWVLVVVGGVLACYSLLVTVAFIIFWVRSKRSRLLHSDFMN<br>MTPRRPGPTRKHYQAYAAPRDFAAYRS*
HPA Bridge<br><br>Dual HPA<br><br>CD28 H/TM<br>Inactivated CD28 ICD	RVQSGKIDCGNDAGWAKVPSDDPGRDNTRELAKNITFASPYCRPPVVLLSITQL<br>DVEQSQNLRVIARLYSVSPTGFKASCYTWHNTKVYSMSISWISIENYGGGGSGG<br>GGSGGGGSRVQSGKIDCGNDAGWAKVPSDDPGRDNTRELAKNITFASPYCRPP<br>VVLLSITQLDVEQSQNLRVIARLYSVSPTGFKASCYTWHNTKVYSMSISWISIEN<br>YIEVMYPPPYLDNEKSNGTIIHVKGKHLCPSPLFPGPSKPFWVLVVVGGVLACY<br>SLLVTVAFIIFWVRSKRSRLLHSDFMNMTPRRPGPTRKHYQAYAAPRDFAAYRS*

Supplemental Table 3. Lectin-based CAR T cells

scFv sequence was replaced by HPA lectins, Siglec-15, MGL. Each lectins were linked by (G4S)3 Linker.

**HPA x1 CAR T cell**<br>CD8 leader<br>Codon Optimized HPA<br>CD8 H/TM<br>4-1BB ICD<br>CD3ζ<br>mCherry	MALPVTALLLPLALLLHAARPRVQSGKIDCGNDAGWAKVPSDDPGRDNTRE<br>LAKNITFASPYCRPPVVLLSITQLDVEQSQNLRVIARLYSVSPTGFKASCYTW<br>HNTKVYSMSISWISIENYTTTPAPRPPTPAPTIASQPLSLRPEACRPAAGGAVH<br>TRGLDFACDIYIWAPLAGTCGVLLLSLVITLYCKRGRKKLLYIFKQPFMRPVQ<br>TTQEEDGCSCRFPEEEEGGCELRVKFSRSADAPAYQQGQNQLYNELNLGRRE<br>EYDVLDKRRGRDPEMGGKPRRKNPQEGLYNELQKDKMAEAYSEIGMKGER<br>RRGKGHDGLYQGLSTATKDTYDALHMQALPPRSGGGGEGRGSLLTCGDVE<br>ENPGPRMVSKGEEDNMAIIKEFMRFKVHMEGSVNGHEFEIEGEGEGRPYEG<br>TQTAKLKVTKGGPLPFAWDILSPQFMYGSKAYVKHPADIPDYLKLSFPEGFK<br>WERVMNFEDGGVVTVTQDSSLQDGEFIYKVKLRGTNFPSDGPVMQKKTMG<br>WEASSERMYPEDGALKGEIKQRLKLKDGGHYDAEVKTTYKAKKPVQLPGA<br>YNVNIKLDITSHNEDYTIVEQYERAEGRHSTGGMDELYK*
**HPA x2 CAR T cell**<br>CD8 leader<br>Codon Optimized HPA<br>$(G_4S)_3$ Linker<br>Codon Optimized HPA<br>CD8 H/TM<br>4-1BB ICD<br>CD3ζ<br>mCherry	MALPVTALLLPLALLLHAARPRVQSGKIDCGNDAGWAKVPSDDPGRDNTRE<br>LAKNITFASPYCRPPVVLLSITQLDVEQSQNLRVIARLYSVSPTGFKASCYTW<br>HNTKVYSMSISWISIENYGGGGSGGGGSGGGGSRVQSGKIDCGNDAGWAK<br>VPSDDPGRDNTRELAKNITFASPYCRPPVVLLSITQLDVEQSQNLRVIARLYS<br>VSPTGFKASCYTWHNTKVYSMSISWISIENYTTTPAPRPPTPAPTIASQPLSLR<br>PEACRPAAGGAVHTRGLDFACDIYIWAPLAGTCGVLLLSLVITLYCKRGRKK<br>LLYIFKQPFMRPVQTTQEEDGCSCRFPEEEEGGCELRVKFSRSADAPAYQQG<br>QNQLYNELNLGRREEYDVLDKRRGRDPEMGGKPRRKNPQEGLYNELQKDK<br>MAEAYSEIGMKGERRRGKGHDGLYQGLSTATKDTYDALHMQALPPRSGGG<br>GEGRGSLLTCGDVEENPGPRMVSKGEEDNMAIIKEFMRFKVHMEGSVNGH<br>EFEIEGEGEGRPYEGTQTAKLKVTKGGPLPFAWDILSPQFMYGSKAYVKHPA<br>DIPDYLKLSFPEGFKWERVMNFEDGGVVTVTQDSSLQDGEFIYKVKLRGTN<br>FPSDGPVMQKKTMGWEASSERMYPEDGALKGEIKQRLKLKDGGHYDAEV<br>KTTYKAKKPVQLPGAYNVNIKLDITSHNEDYTIVEQYERAEGRHSTGGMDE<br>LYK*
**HPA x3 CAR T cell**<br>CD8 leader	MALPVTALLLPLALLLHAARPRVQSGKIDCGNDAGWAKVPSDDPGRDNTRE<br>LAKNITFASPYCRPPVVLLSITQLDVEQSQNLRVIARLYSVSPTGFKASCYTW<br>HNTKVYSMSISWISIENYGGGGSGGGGSGGGGSRVQSGKIDCGNDAGWAK<br>VPSDDPGRDNTRELAKNITFASPYCRPPVVLLSITQLDVEQSQNLRVIARLYS

Codon Optimized HPA<br><br>$(G_4S)_3$ Linker<br><br>Codon Optimized HPA<br><br>CD8 H/TM<br><br>4-1BB ICD<br><br>CD3ζ<br><br>mCherry	VSPTGFKASCYTWHNTKVYSMSISWISIENYGGGGSGGGGSGGGGSRVQSG<br>KIDCGNDAGWAKVPSDDPGRDNTRELAKNITFASPYCRPPVVLLSITQLDVE<br>QSQNLRVIARLYSVSPTGFKASCYTWHNTKVYSMSISWISIENYTTTPAPRPP<br>TPAPTIASQPLSLRPEACRPAAGGAVHTRGLDFACDIYIWAPLAGTCGVLLLS<br>LVITLYCKRGRKKLLYIFKQPFMRPVQTTQEDGCSCRFPEEEEGGCELRVKF<br>SRSADAPAYQQGQNQLYNELNLGRREEYDVLDKRRGRDPEMGGKPRRKNP<br>QEGLYNELQKDKMAEAYSEIGMKGERRRGKGHDGLYQGLSTATKDTYDAL<br>HMQALPPRSGGGGEGRGSLLTCGDVEENPGPRMVSKGEEDNMAIIKEFMRF<br>KVHMEGSVNGHEFEIEGEGEGRPYEGTQTAKLKVTKGGPLPFAWDILSPQF<br>MYGSKAYVKHPADIPDYLKLSFPEGFKWERVMNFEDGGVVTVTQDSSLQD<br>GEFIYKVKLRGTNFPSDGPVMQKKTMGWEASSERMYPEDGALKGEIKQRL<br>KLKDGGHYDAEVKTTYKAKKPVQLPGAYNVNIKLDITSHNEDYTIVEQYER<br>AEGRHSTGGMDELYK*
**MGLx1 CAR T cell**<br><br>CD8 leader<br><br>MGLx1<br><br>CD8 H/TM<br><br>4-1BB ICD<br><br>CD3ζ<br><br>mCherry	MALPVTALLLPLALLLHAARPQNSKFQRDLVTLRTDFSNFTSNTVAEIQALTS<br>QGSSLEETIASLKAEVEGFKQERQAGVSELQEHTTQKAHLGHCPHCPSVCVP<br>VHSEMLLRVQQLVQDLKKLTCQVATLNNNASTEGTCCPVNWVEHQDSCYW<br>FSHSGMSWAEAEKYCQLKNAHLVVINSREEQNFVQKYLGSAYTWMGLSDP<br>EGAWKWVDGTDYATGFQNWKPGQPDDWQGHGLGGGEDCAHFHPDGRWN<br>DDVCQRPYHWVCEAGLGQTSQESHTTTPAPRPPTPAPTIASQPLSLRPEACRP<br>AAGGAVHTRGLDFACDIYIWAPLAGTCGVLLLSLVITLYCKRGRKKLLYIFK<br>QPFMRPVQTTQEDGCSCRFPEEEEGGCELRVKFSRSADAPAYQQGQNQLY<br>NELNLGRREEYDVLDKRRGRDPEMGGKPRRKNPQEGLYNELQKDKMAEA<br>YSEIGMKGERRRGKGHDGLYQGLSTATKDTYDALHMQALPPRSGGGGEGR<br>GSLLTCGDVEENPGPRMVSKGEEDNMAIIKEFMRFKVHMEGSVNGHEFEIE<br>GEGEGRPYEGTQTAKLKVTKGGPLPFAWDILSPQFMYGSKAYVKHPADIPD<br>YLKLSFPEGFKWERVMNFEDGGVVTVTQDSSLQDGEFIYKVKLRGTNFPSD<br>GPVMQKKTMGWEASSERMYPEDGALKGEIKQRLKLKDGGHYDAEVKTTY<br>KAKKPVQLPGAYNVNIKLDITSHNEDYTIVEQYERAEGRHSTGGMDELYK*
**MGLx2 CAR T cell**<br><br>CD8 leader<br><br>MGLx1<br><br>$(G_4S)_3$ Linker<br><br>MGLx1<br><br>CD8 H/TM	MALPVTALLLPLALLLHAARPQNSKFQRDLVTLRTDFSNFTSNTVAEIQALTS<br>QGSSLEETIASLKAEVEGFKQERQAGVSELQEHTTQKAHLGHCPHCPSVCVP<br>VHSEMLLRVQQLVQDLKKLTCQVATLNNNASTEGTCCPVNWVEHQDSCYW<br>FSHSGMSWAEAEKYCQLKNAHLVVINSREEQNFVQKYLGSAYTWMGLSDP<br>EGAWKWVDGTDYATGFQNWKPGQPDDWQGHGLGGGEDCAHFHPDGRWN<br>DDVCQRPYHWVCEAGLGQTSQESHGGGGSGGGGSGGGGSQNSKFQRDLV<br>TLRTDFSNFTSNTVAEIQALTSQGSSLEETIASLKAEVEGFKQERQAGVSELQ<br>EHTTQKAHLGHCPHCPSVCVPVHSEMLLRVQQLVQDLKKLTCQVATLNNNA<br>STEGTCCPVNWVEHQDSCYWFSHSGMSWAEAEKYCQLKNAHLVVINSREE<br>QNFVQKYLGSAYTWMGLSDPEGAWKWVDGTDYATGFQNWKPGQPDDWQ<br>GHGLGGGEDCAHFHPDGRWNDDVCQRPYHWVCEAGLGQTSQESHTTTPAP<br>RPPTPAPTIASQPLSLRPEACRPAAGGAVHTRGLDFACDIYIWAPLAGTCGVL<br>LLSLVITLYCKRGRKKLLYIFKQPFMRPVQTTQEDGCSCRFPEEEEGGCELR

4-1BB ICD<br><br>CD3ζ<br><br>mCherry	VKFSRSADAPAYQQGQNQLYNELNLGRREEYDVLDKRRGRDPEMGGKPRR<br>KNPQEGLYNELQKDKMAEAYSEIGMKGERRRGKGHDGLYQGLSTATKDTY<br>DALHMQALPPRSGGGGEGRGSLLTCGDVEENPGPRMVSKGEEDNMAIIKEF<br>MRFKVHMEGSVNGHEFEIEGEGEGRPYEGTQTAKLKVTKGGPLPFAWDILS<br>PQFMYGSKAYVKHPADIPDYLKLSFPEGFKWERVMNFEDGGVVTVTQDSSL<br>QDGEFIYKVKLRGTNFPSDGPVMQKKTMGWEASSERMYPEDGALKGEIKQ<br>RLKLKDGGHYDAEVKTTYKAKKPVQLPGAYNVNIKLDITSHNEDYTIVEQY<br>ERAEGRHSTGGMDELYK*
**Siglec-15x1 CAR T cell**<br><br>CD8 leader<br><br>Siglec-15 Phe20-Thr263<br><br>CD8 H/TM<br><br>4-1BB ICD<br><br>CD3ζ<br><br>mCherry	MALPVTALLLPLALLLHAARPFVRTKIDTTENLLNTEVHSSPAQRWSMQVPP<br>EVSAEAGDAAVLPCTFTHPHRHYDGPLTAIWRAGEPYAGPQVFRCAAARGS<br>ELCQTALSLHGRFRLLGNPRRNDLSLRVERLALADDRRYFCRVEFAGDVHD<br>RYESRHGVRLHVTAAPRIVNISVLPGPAHAFRALCTAEGEPPPALAWSGQAL<br>GNSLAAVRSPREGHGHLVTAELPALTHDGRYTCTAANSLGRSEASVYLFRFH<br>GASGASTTTTPAPRPPTPAPTIASQPLSLRPEACRPAAGGAVHTRGLDFACDIY<br>IWAPLAGTCGVLLLSLVITLYCKRGRKKLLYIFKQPFMRPVQTTQEEDGCSCR<br>FPEEEEGGCELRVKFSRSADAPAYQQGQNQLYNELNLGRREEYDVLDKRRG<br>RDPEMGGKPRRKNPQEGLYNELQKDKMAEAYSEIGMKGERRRGKGHDGLY<br>QGLSTATKDTYDALHMQALPPRSGGGGEGRGSLLTCGDVEENPGPRMVSK<br>GEEDNMAIIKEFMRFKVHMEGSVNGHEFEIEGEGEGRPYEGTQTAKLKVTK<br>GGPLPFAWDILSPQFMYGSKAYVKHPADIPDYLKLSFPEGFKWERVMNFED<br>GGVVTVTQDSSLQDGEFIYKVKLRGTNFPSDGPVMQKKTMGWEASSERMY<br>PEDGALKGEIKQRLKLKDGGHYDAEVKTTYKAKKPVQLPGAYNVNIKLDIT<br>SHNEDYTIVEQYERAEGRHSTGGMDELYK*
**Siglec-15x2 CAR T cell**<br><br>CD8 leader<br><br>Siglec-15 Phe20-Thr263<br><br>$(G_4S)_3$ Linker<br><br>Siglec-15 Phe20-Thr263<br><br>CD8 H/TM<br><br>4-1BB ICD<br><br>CD3ζ<br><br>mCherry	MALPVTALLLPLALLLHAARPFVRTKIDTTENLLNTEVHSSPAQRWSMQVPP<br>EVSAEAGDAAVLPCTFTHPHRHYDGPLTAIWRAGEPYAGPQVFRCAAARGS<br>ELCQTALSLHGRFRLLGNPRRNDLSLRVERLALADDRRYFCRVEFAGDVHD<br>RYESRHGVRLHVTAAPRIVNISVLPGPAHAFRALCTAEGEPPPALAWSGQAL<br>GNSLAAVRSPREGHGHLVTAELPALTHDGRYTCTAANSLGRSEASVYLFRFH<br>GASGASTGGGGSGGGGSGGGGSFVRTKIDTTENLLNTEVHSSPAQRWSMQV<br>PPEVSAEAGDAAVLPCTFTHPHRHYDGPLTAIWRAGEPYAGPQVFRCAAAR<br>GSELCQTALSLHGRFRLLGNPRRNDLSLRVERLALADDRRYFCRVEFAGDVH<br>DRYESRHGVRLHVTAAPRIVNISVLPGPAHAFRALCTAEGEPPPALAWSGQA<br>LGNSLAAVRSPREGHGHLVTAELPALTHDGRYTCTAANSLGRSEASVYLFRF<br>HGASGASTTTTPAPRPPTPAPTIASQPLSLRPEACRPAAGGAVHTRGLDFACDI<br>YIWAPLAGTCGVLLLSLVITLYCKRGRKKLLYIFKQPFMRPVQTTQEEDGCS<br>CRFPEEEEGGCELRVKFSRSADAPAYQQGQNQLYNELNLGRREEYDVLDKR<br>RGRDPEMGGKPRRKNPQEGLYNELQKDKMAEAYSEIGMKGERRRGKGHD<br>GLYQGLSTATKDTYDALHMQALPPRSGGGGEGRGSLLTCGDVEENPGPRMV<br>SKGEEDNMAIIKEFMRFKVHMEGSVNGHEFEIEGEGEGRPYEGTQTAKLKV<br>TKGGPLPFAWDILSPQFMYGSKAYVKHPADIPDYLKLSFPEGFKWERVMNFE<br>DGGVVTVTQDSSLQDGEFIYKVKLRGTNFPSDGPVMQKKTMGWEASSERM<br>YPEDGALKGEIKQRLKLKDGGHYDAEVKTTYKAKKPVQLPGAYNVNIKLDI<br>TSHNEDYTIVEQYERAEGRHSTGGMDELYK*
