# Supplementary material for: Tuning CAR-T cells by targeting cancer-associated glycan in pancreatic cancer
Source: Nat Commun. 2025 Dec 10;16:11246. doi: 10.1038/s41467-025-66102-2 (PMC12717153; doi:10.1038/s41467-025-66102-2)
Supplement: Supplementary file 2 — Reporting Summary [file 41467_2025_66102_MOESM2_ESM.pdf]

nature portfolio

Corresponding author(s): Marcela V. Maus

Last updated by author(s): 10/14/2025

Reporting Summary

Nature Portfolio wishes to improve the reproducibility of the work that we publish. This form provides structure for consistency and transparency in reporting. For further information on Nature Portfolio policies, see our Editorial Policies and the Editorial Policy Checklist.

Statistics

For all statistical analyses, confirm that the following items are present in the figure legend, table legend, main text, or Methods section.

n/a	Confirmed	
☐	☒	The exact sample size (*n*) for each experimental group/condition, given as a discrete number and unit of measurement
☐	☒	A statement on whether measurements were taken from distinct samples or whether the same sample was measured repeatedly
☐	☒	The statistical test(s) used AND whether they are one- or two-sided<br>*Only common tests should be described solely by name; describe more complex techniques in the Methods section.*
☒	☐	A description of all covariates tested
☐	☒	A description of any assumptions or corrections, such as tests of normality and adjustment for multiple comparisons
☐	☒	A full description of the statistical parameters including central tendency (e.g. means) or other basic estimates (e.g. regression coefficient) AND variation (e.g. standard deviation) or associated estimates of uncertainty (e.g. confidence intervals)
☐	☒	For null hypothesis testing, the test statistic (e.g. *F*, *t*, *r*) with confidence intervals, effect sizes, degrees of freedom and *P* value noted<br>*Give P values as exact values whenever suitable.*
☒	☐	For Bayesian analysis, information on the choice of priors and Markov chain Monte Carlo settings
☒	☐	For hierarchical and complex designs, identification of the appropriate level for tests and full reporting of outcomes
☒	☐	Estimates of effect sizes (e.g. Cohen's *d*, Pearson's *r*), indicating how they were calculated

Our web collection on statistics for biologists contains articles on many of the points above.

Software and code

Policy information about availability of computer code

Data collection	BD Fortessa X-20 flow cytometry, NovoCyte Penteon Flow Cytometer (Agilent), and MA900 Cell Sorter (Sony) were used for flow cytometry data acquisition. A Biotek Neo2 luminescence plate reader was used for luciferase killing assay. Fluorescence images of cells were acquired using Zeiss LSM 780 confocal microscope and Incucyte SX5 (Sartorius). Live-cell killing assay were acquired using Incucyte SX5 (Sartorius). Cell avidity assay were acquired using z-Movi Cell Avidity Analyzer (Lumicks). A Fisher Scientific Nanodrop 2000C was used to characterize DNAs. A BioRad T100 Thermocycler was used to do PCR for cloning.
Data analysis	Microsoft Excel for Microsoft 365 MSO (Version 2307), GraphPad Prism (v10), MATLAB R2019a, ImageJ (v1.53s) were used for data analysis. FlowJo (v10) was used for flow cytometry data analysis. Ocean software (version 1.5.5, Lumicks) for cell avidity analysis. QuPath (v0.5.1) was used for IHC data analysis.

For manuscripts utilizing custom algorithms or software that are central to the research but not yet described in published literature, software must be made available to editors and reviewers. We strongly encourage code deposition in a community repository (e.g. GitHub). See the Nature Portfolio guidelines for submitting code & software for further information.

Data

Policy information about availability of data

All manuscripts must include a data availability statement. This statement should provide the following information, where applicable:

- Accession codes, unique identifiers, or web links for publicly available datasets
- A description of any restrictions on data availability
- For clinical datasets or third party data, please ensure that the statement adheres to our policy

All data shown in this manuscript are provided as source data files. All requests for raw and analyzed data will be reviewed by MGH to determine whether they are subject to intellectual property or confidentiality obligations. Any data and materials that can be shared will be released using a material transfer agreement. Please contact M.V. Maus at mvmaus@mgh.harvard.edu

Research involving human participants, their data, or biological material

Policy information about studies with human participants or human data. See also policy information about sex, gender (identity/presentation), and sexual orientation and race, ethnicity and racism.

Reporting on sex and gender	N/A - Our studies are not classified as human subject research according to the definitions of the National Institutes of Health; All human biospecimens (e.g. blood for immune cell isolation) were obtained as de-identified products
Reporting on race, ethnicity, or other socially relevant groupings	N/A
Population characteristics	N/A
Recruitment	N/A
Ethics oversight	Anonymized human blood samples were used with approval of the Institutional Review Board (IRB) at the Massachusetts General Hospital (MGH) and declared as non-human subjects research.

Note that full information on the approval of the study protocol must also be provided in the manuscript.

Field-specific reporting

Please select the one below that is the best fit for your research. If you are not sure, read the appropriate sections before making your selection.

[x] Life sciences [ ] Behavioural & social sciences [ ] Ecological, evolutionary & environmental sciences

For a reference copy of the document with all sections, see nature.com/documents/nr-reporting-summary-flat.pdf

Life sciences study design

All studies must disclose on these points even when the disclosure is negative.

Sample size	Sample sizes were based on typical sizes in the field; statistical methods were not used to pre-determine sample sizes. In mouse experiment, sample sizes (n = 5 per group) were determined based on power calculations and prior experience with similar in vivo experiments, which were sufficient to detect biologically meaningful differences.
Data exclusions	Mice that died before CAR T-cell infusion or exhibited extreme values inconsistent with biological replicates were excluded from the analysis. No other data were excluded.
Replication	Multiple biological and/or experimental replicates were conducted as noted in the figure legends.
Randomization	Cells were selected at random for all single-cell analyses. For in vivo studies, mice were randomized to treatment groups prior to T cell transfer to normalize starting tumor burden across groups.
Blinding	For in vivo experiments, one veterinary technician performed all experiments blinded to expected outcomes. Researchers were not blinded to treatment groups for in vitro studies because knowledge of this information was essential to conduct the studies.

Reporting for specific materials, systems and methods

We require information from authors about some types of materials, experimental systems and methods used in many studies. Here, indicate whether each material, system or method listed is relevant to your study. If you are not sure if a list item applies to your research, read the appropriate section before selecting a response.

Materials & experimental systems

n/a	Involved in the study	
☐	☒	Antibodies
☐	☒	Eukaryotic cell lines
☒	☐	Palaeontology and archaeology
☐	☒	Animals and other organisms
☒	☐	Clinical data
☒	☐	Dual use research of concern
☒	☐	Plants

Methods

n/a	Involved in the study	
☒	☐	ChIP-seq
☐	☒	Flow cytometry
☒	☐	MRI-based neuroimaging

Antibodies

Antibodies used	Alexa Fluor 647 conjugated FluoTag-X2 anti-ALFA (N1502-AF647-L; NanoTag Biotechnologies)<br>Alexa Fluor 647 conjugated G4S (E7O2V) antibody (69782S; Cell Signaling Technology)<br>Alexa Fluor 700 conjugated anti-human CD3 antibody (300424; BioLegend)<br>Per/Cy7 conjugated anti-human CD4 antibody (300518; BioLegend)<br>PerCP conjugated anti-human CD8α antibody (301032; BioLegend)<br>FITC conjugated anti-human CCR7 antibody (561271; BD BioSciences)<br>Brilliant Violet 42 conjugated anti-human CD45RA antibody (304130; BioLegend)<br>Alexa Fluor 700 conjugated anti-human CD45 antibody (304024; BioLegend)<br>Alexa Fluor 647 conjugated HPA lectin (L32454; Thermo Scientific)<br>PE conjugated CD19 (363003; BioLegend)<br>APC conjugated anti-human CD69 (310910; BioLegend)<br>anti-human Tn-Muc1 (FHD14210-100; ProteoGenix)<br>Alexa Fluor 647 conjugated goat anti-human IgG (H+L) recombinant secondary antibody (A56019; Invitrogen)<br>Muc1 (NBP2-47883-0.1mg; Novus Biologicals)<br>anti-human Tn-MUC1 (FHD14210-100; ProteoGenix)<br>Tn-antigen (GTX82968; GeneTex)<br>Tn antigen (SBH-Tn-100ug, SBH Sciences)
Validation	All antibodies were obtained from commercial sources which provided authentication sheets. When possible, antibodies were additionally validated by confirming correct sub-cellular localization in immunolabeled cells and reactivity via flow cytometry.

Eukaryotic cell lines

Policy information about cell lines and Sex and Gender in Research

Cell line source(s)	Capan-2 (HTB-80), RPMI 8226 (CCL-155), HEK293T (CRL-3216) and Jurkat (TIB-152) were obtained from the American Tissue Culture Collection (ATCC). An unimmortalized PDX PDAC cell line, PDX 1294, PDX1275, PDX1319, were kindly provided by the Liss laboratory at Massachusetts General Hospital (MGH)
Authentication	All cell lines except PDXs were obtained as authenticated products from ATCC.
Mycoplasma contamination	All cell lines were regularly tested and confirmed negative for mycoplasma infection
Commonly misidentified lines (See ICLAC register)	No commonly misidentified cells were used in this study.

Animals and other research organisms

Policy information about studies involving animals; ARRIVE guidelines recommended for reporting animal research, and Sex and Gender in Research

Laboratory animals	Male and female NOD-SCID-γ chain −/− (NSG) mice (6-12 weeks) were used for all experiments.
Wild animals	Wild animals were not used in this study.
Reporting on sex	Male and female mice were used in this study and findings were not specific to one sex.
Field-collected samples	There were no field samples collected for this study.
Ethics oversight	Experiments were performed according to protocols approved by the MGH Institutional Animal Care and Use Committee (IACUC).

Note that full information on the approval of the study protocol must also be provided in the manuscript.

Plants

Seed stocks	*Report on the source of all seed stocks or other plant material used. If applicable, state the seed stock centre and catalogue number. If plant specimens were collected from the field, describe the collection location, date and sampling procedures.*
Novel plant genotypes	*Describe the methods by which all novel plant genotypes were produced. This includes those generated by transgenic approaches, gene editing, chemical/radiation-based mutagenesis and hybridization. For transgenic lines, describe the transformation method, the number of independent lines analyzed and the generation upon which experiments were performed. For gene-edited lines, describe the editor used, the endogenous sequence targeted for editing, the targeting guide RNA sequence (if applicable) and how the editor was applied.*
Authentication	*Describe any authentication procedures for each seed stock used or novel genotype generated. Describe any experiments used to assess the effect of a mutation and, where applicable, how potential secondary effects (e.g. second site T-DNA insertions, mosiacism, off-target gene editing) were examined.*

Flow Cytometry

Plots

Confirm that:

☒ The axis labels state the marker and fluorochrome used (e.g. CD4-FITC).

☒ The axis scales are clearly visible. Include numbers along axes only for bottom left plot of group (a 'group' is an analysis of identical markers).

☒ All plots are contour plots with outliers or pseudocolor plots.

☒ A numerical value for number of cells or percentage (with statistics) is provided.

Methodology

Sample preparation	Sample preparation is described in the methods
Instrument	BD Fortessa X-20 flow cytometry, NovoCyte Penteon Flow Cytometer (Agilent), and MA900 Cell Sorter (Sony)
Software	The FlowJo V10 software package was used for data analysis
Cell population abundance	The samples were more than 95% pure as determined by post-sort flow-cytometry analysis
Gating strategy	Examples and descriptions for all gating strategies are in the Supplementary Information. FSC-A/SSC-A plot was used to gate for the starting cell population. Singlets were selected with SSC-H/SSC-A plots. Live cells were selected with SSC-A/DAPI+ plots.

☒ Tick this box to confirm that a figure exemplifying the gating strategy is provided in the Supplementary Information.
